# A prospective, randomized trial of liposomal bupivacaine compared to conventional bupivacaine on pain control and postoperative opioid use in adults receiving adductor canal blocks for total knee arthroplasty

**DOI:** 10.1186/s42836-023-00226-y

**Published:** 2024-02-01

**Authors:** Aurora Quaye, Brian McAllister, Joseph R. Garcia, Orion Nohr, Sarah J. Laduzenski, Lucy Mack, Christine R. Kerr, Danielle A. Kerr, Charonne N. Razafindralay, Janelle M. Richard, Wendy Y. Craig, Stephen Rodrigue

**Affiliations:** 1 Department of Anesthesiology, Northern Light Mercy Hospital, 175 Fore River Parkway, Portland, ME 04102 USA; 2 Department of Anesthesiology and Perioperative Medicine, Maine Medical Center, 22 Bramhall St, Portland, ME 04102 USA; 3 Division of Anesthesiology, Spectrum Healthcare Partners, 324 Gannett Drive, Suite 200, South Portland, ME 04106 USA; 4 Tufts University School of Medicine, 145 Harrison Ave, Boston, MA 02111 USA; 5 University of New England College of Osteopathic Medicine, 11 Hills Beach Rd, Biddeford, ME 04005 USA; 6 Maine Health Institute for Research, 81 Research Dr, Scarborough, ME 04074 USA; 7 Northern Light Mercy Orthopedics, 20 Northbrook Dr, Falmouth, ME 04105 USA

**Keywords:** Regional anesthesia, Nerve block, Arthroplasty, Replacement, Knee, Pain, Postoperative, Anesthetics, Local

## Abstract

**Background:**

Total knee arthroplasty (TKA) is a commonly performed procedure to alleviate pain and improve functional limitations caused by end-stage joint damage. Effective management of postoperative pain following TKA is crucial to the prevention of complications and enhancement of recovery. Adductor canal blocks (ACB) with conventional bupivacaine (CB) provide adequate analgesia after TKA, but carry a risk of rebound pain following block resolution. Liposomal bupivacaine (LB) is an extended-release local anesthetic that can provide up to 72 h of pain relief. The objective of this study was to compare postoperative outcomes between ACBs using LB and CB after TKA.

**Methods:**

This single institution, prospective, randomized, clinical trial enrolled patients scheduled for TKA. Participants were randomized to receive ACB with either LB or CB. Pain scores up to 72 h postoperatively were assessed as the primary outcome. Opioid consumption and length of stay were evaluated as secondary outcomes.

**Results:**

A total of 80 patients were enrolled. Demographic and clinical characteristics were similar between the two groups. LB group showed significantly lower cumulative opioid use during the 72 h evaluated (*P* = 0.016). There were no differences in pain scores or length of stay between the groups.

**Conclusion:**

The study demonstrated that LB ACBs led to significantly lower opioid consumption in the days following TKA without affecting pain scores or length of stay. This finding has important implications for improving postoperative outcomes and reducing opioid use in TKA patients. Previous studies have reported inconsistent results regarding the benefits of LB, highlighting the need for further research.

**Trial registration:**

This project was retrospectively registered with clinicaltrials.gov (NCT05635916) on 2 December 2022.

**Supplementary Information:**

The online version contains supplementary material available at 10.1186/s42836-023-00226-y.

## Background

Total knee arthroplasty (TKA) is a procedure most commonly performed to ameliorate severe pain and improve the functional limitations characteristic of end-stage joint damage [1]. Managing post-TKA pain can be quite challenging, and inadequate postoperative pain relief can lead to impaired mobility, pain-related distress, sleep disturbances, and persistent opioid use [2].

Regional anesthesia, in the form of femoral nerve and adductor canal nerve blocks (ACB), is used to manage pain control following TKA [3]. Femoral nerve blocks were the first peripheral nerve block employed for post-surgical analgesia. However, they are now used less frequently since blockade of the motor branches of this nerve leads to quadriceps weakness and increased fall risk after surgery [4]. Additionally, ambulation after surgery can be significantly delayed with femoral nerve blocks [5]. ACBs, performed at the mid-thigh region where the sensory neuronal branches of the saphenous nerve are located, do not affect the motor branches of the femoral nerve and quadriceps muscle strength is preserved [6–8]. Thus, ACB is considered a superior method for post-surgical analgesia [9]. ACBs have been studied extensively and have shown significant benefits compared to opioid-based analgesia in improving participation in physical therapy, decreasing opioid consumption, and improving postoperative patient satisfaction [10].

As an interfacial plane block, ACBs can be performed as a single injection or as a continuous infusion via the insertion of a catheter, with respective advantages and disadvantages [11]. Continuous peripheral nerve blocks can provide prolonged postoperative analgesia; however, they can be time-consuming and resource-intensive and are associated with several complications, including catheter infection, obstructions, and fluid leakage [12]. Additionally, catheter displacement can be frequent with ACBs, since the aponeurosis where the saphenous nerve lies is superficial, leading to easy dislodgement and block failure [5, 13]. A single perineural injection of local anesthetic is simpler to place, more cost-effective, and has a lower infection risk; however, the risk of rebound pain is higher when conventional bupivacaine is used [14].

Liposomal bupivacaine (LB), an extended-release local anesthetic that provides analgesia for up to 72 h, potentially combines the benefits of a single injection nerve block with the prolonged effects of continuous catheter infusions [15]. In the United States, LB has Food and Drug Administration approval for post-surgical anesthesia via local infiltration and interscalene brachial plexus blocks [16]. It is not yet approved for ACBs, although off-label use has been reported [17–19]. Although a meta-analysis concluded that, overall, liposomal bupivacaine is not superior clinically to conventional bupivacaine when used in peripheral nerve blocks, it may improve outcome metrics when used in ACB for knee surgery [18, 20, 21].

The purpose of our study was to compare differences in postoperative outcomes between ACBs performed using liposomal bupivacaine vs. conventional bupivacaine after TKA. Our primary outcome measure was pain scores during the first 72 h following surgery. Secondary outcomes included opioid consumption and inpatient length of stay. The findings of this study further inform guidance regarding the utility of liposomal bupivacaine ACBs for total knee arthroplasty.

## Methods

This was a prospective, randomized, single-blind clinical trial comparing ACBs using liposomal bupivacaine or conventional bupivacaine among patients scheduled for TKA. The study was reviewed and approved by the Mercy Hospital Institutional Review Board and registered with clinicaltrials.gov (NCT05635916). Patient enrollment began on 27 September 2022 and ended on 11 April 2023. Inclusion criteria for patients undergoing primary TKA were: (1) age 18–79 years and (2) an American Society of Anesthesiologists (ASA) classification between I–III. Patients were excluded if they were unable to tolerate local anesthetics, had a body mass index (BMI) of greater than 40 kg/m^2^, had a history of being diagnosed with substance use disorder, had a baseline opioid use of greater than 90 morphine milligram equivalents (MME)/day, or were scheduled for revision arthroplasty.

### Study protocol

Patients were notified about the study during their preoperative clinical appointment and informed consent was obtained by an anesthesiologist on the study team in person up to the day of surgery. Following informed consent, patients were randomized into the two treatment arms at a 1:1 ratio using NQuery Software (Statistical Solutions, Boston, MA, USA). The randomization sequence was generated by the data scientist on the research team and randomization assignments were kept in sequentially-numbered opaque envelopes. Participants were randomized to receive either (i) ACB with injection of 10 cc 13.3% liposomal bupivacaine combined with 10 cc 0.25% bupivacaine within 1 h following surgery or (ii) ACB with injection of 20 cc 0.5% bupivacaine within 1 h following surgery. The anesthesiologist performing the ACB was aware of the participant’s assignment. However, the surgery team, patient, and staff collecting data were blinded.

All participants received similar multi-modal pain management strategies consisting of ketamine, opioids, non-steroidal anti-inflammatories, and acetaminophen from the time of the surgical procedure to postoperative day (POD) 2. Additionally, pericapsular local anesthetic infiltration was performed by the surgical team intraoperatively for both patient cohorts. All multimodal agents were recorded for analysis.

Ultrasound-guided nerve blocks targeting the saphenous nerve were performed within one hour following the surgical procedure in the postoperative care unit (PACU). Patients were provided with opioid rescue agents in the form of tramadol, oxycodone, or hydromorphone to ensure that post-surgical pain was adequately controlled. All opioid medications were converted to MMEs for analysis. Additionally, pain scores were recorded from time to arrival in PACU to POD-2. Pain scores were measured using a numeric rating scale (NRS) (0 = no pain; 10 = worst possible pain). “Worst”, “average” and “least” pain scores were assessed for the preceding time interval at 24 h, 48 h, and 72 h following surgery. If a participant was discharged prior to 72 h after surgery, they were given a log to record their pain scores and pain medication use and reported these data to study staff via telephone. Additional outcomes recorded were the type of anesthesia used intraoperatively, block performance time, length time of PACU, and overall length of hospital stay.

### Sample size

 We estimated the study size based on postoperative pain scores in a recent study of surgical patients at our institution which yielded a mean NRS of 5 ± 2.8 [22]. Our estimate for effect size is based on published data showing that a difference in NRS of two points is clinically significant [23]. A sample size of 32 per group will have 80% power to detect a difference in means of two (the difference between a Group 1 mean, μ 1, of 5.0 and a Group 2 mean, μ 2, of 3.0) assuming the common standard deviation is 2.8 using a two group* t*-test with a 0.05 significance level. To account for dropout, we enrolled an additional 8 patients for each group for a total sample size of 80 patients.

### Statistical analysis

We performed our primary data analysis and interpreted findings in the intention-to-treat population and performed descriptive statistics only in the per-protocol population. We summarized data as frequency (*n*, %) for categorical data and as mean and standard deviation or as median and interquartile range, as appropriate, for continuous variables. We compared data between the two treatment arms using chi-square or Fisher’s exact test (categorical data) and *t*-tests or Mann–Whitney *u*-tests (continuous data), as appropriate. We examined differences in opioid dose over time among those receiving an opioid by repeated measures analysis using a linear mixed effects model after log^10^ transformation of opioid dose data. We performed this analysis over postoperative days 0–2, defined as sequential 24 h periods following discharge from the PACU. Data collected in the PACU were analyzed separately as patients received the ACB in this unit and were still recovering from intraoperative anesthesia. To illustrate the effect of treatment arm on the combination of opioid dose and usage frequency we used repeated measures ANOVA to calculate the estimated marginal mean and standard error of opioid dose (MME) in each time period. For this calculation, we included those receiving no opioids and entered their dose as 0 MME. The skewed distributions of these opioid data precluded full statistical analysis using this method. Data were analyzed using SPSS statistical software version 29 (IBM SPSS Inc, Armonk, NY, USA). Significance was accepted at *P* < 0.05.

## Results

**Fig. 1:**
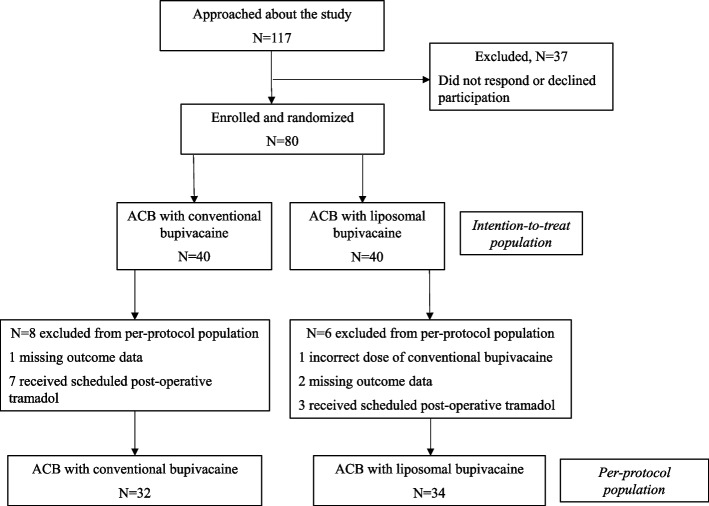
CONSORT diagram

We enrolled a total of 80 patients (40 in each arm) for the intention-to-treat analysis (Fig. 1), of whom 66 (82%) completed the study with no protocol deviations. Table 1 shows that demographic, clinical and intraoperative anesthesia characteristics were similar between the two treatment arms of the study.

**Table 1:** Demographic and clinical characteristics of the study group, stratified by treatment arm

Variable^a^	Bupivacaine type used in adductor canal block (treatment arm)	
	Liposomal^b^	Conventional
N	40	40
Age (years)	68.0 ± 9.0	69.1 ± 8.8
BMI (kg/m^2^)	29.8 ± 4.6	28.8 ± 5.6
Male sex	20 (50.0%)	16 (40.0%)
Race/ethnicity		
White	39 (97.5%)	40 (100.0%)
Black or African American	1 (2.5%)	0 (0.0%)
Active Smoking	2 (5.0%)	1 (2.5%)
ASA class		
1	2 (5.0%)	1 (2.5%)
2	29 (72.5%)	33 (82.5%)
3	9 (22.5%)	6 (15.0%)
Comorbidities		
Depression	10 (25.0%)	16 (40.0%)
Anxiety/PTSD	12 (30.0%)	10 (25%)
Chronic pain	2 (5.0%)	4 (10.0%)
Fibromyalgia	1 (2.5%)	0 (0.0%)
Pre-operative medications		
Opioids < 90 MME/day	2 (5.0%)	1 (2.5%)
Antidepressants	7 (17.5%)	12 (30.0%)
Anti-anxiety	4 (10.0%)	1 (2.5%)
Gabapentin	1 (2.5%)	1 (2.5%)
Initial anesthesia type		
General	3 (7.5%)	1 (2.5%)
Spinal	37 (92.5%) ^c^	39 (97.5%)
Intraoperative pain management		
Opioids		
Received intraoperative opioids, *n* (%)	10 (25.0%)	10 (25.0%)
Total opioid dose if received (MME)	30 (15–47.5)	22.5 (10–32.5)
Non-opioids		
Received ketorolac, *n* (%)	2 (5.0%)	0 (0.0%)
Ketorolac dose if received (mg)	15, 15	None
Ketamine		
Received ketamine, *n* (%)	25 (62.5%)	24 (60.0%)
Ketamine dose if received (mg)	40 (22.5–50)	50 (31.9–50)
Time to perform block (min)	2.2 ± 0.4	2.0 ± 0.2
Duration of anesthesia (h)	1.6 ± 0.2	1.6 ± 0.2

*ASA* American Society of Anesthesiologists, *PTSD* post-traumatic stress disorder, *MME* morphine milligram equivalents

^a^Data shown as *n* (%) or as mean ± standard deviation

^b^One patient received an adductor canal block with liposomal bupivacaine and 0.5% (instead of 0.25%) conventional bupivacaine

^c^One patient was subsequently converted to general anesthesia, as could not place spinal

**Fig. 2:**
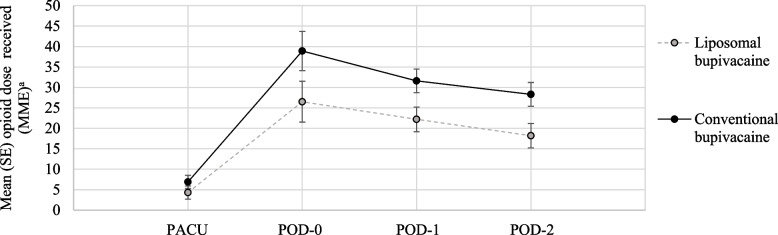
Overall opioid dose (MME), by time period after total knee arthroplasty, stratified by ACB bupivacaine type ACB, adductor canal block; MME, milligram morphine equivalents; PACU, post-anesthesia care unit; POD, postoperative day (defined as consecutive 24 h periods following discharge from the PACU); SE, standard error. ^a^ Mean (SE) opioid dose at each time point was estimated by repeated measures ANOVA; MME was entered as zero when a participant did not receive opioids

We explored the relationship between treatment-arm opioid consumption in two phases, first examining the frequency of use and then the dose among those receiving opioids. Table 2 addresses the frequency of opioid use during the study in the two treatment arms. There was no significant difference in the frequency of opioid use in the PACU or on POD-0 and POD-1. However, on POD-2 opioid use was significantly less frequent in the LB group compared with the CB group (66.7% and 89.5% respectively, *P* = 0.024). Among those patients receiving opioids for postoperative pain management, the time course of daily dose differed significantly between the two treatment arms between POD-0 and POD-2 (*P* = 0.016), with the largest differences in median dose on POD-0 (11.3 MME) and POD-1 (8.5 MME), decreasing to 1.2 MME on POD-2 (Table 2). The Fig. 2 shows the overall time course of opioid consumption in the two treatment arms by including participants receiving no opioids in the estimation of daily opioid dose and illustrates the combined impact of reduced dose and reduced frequency of use during the study period.

**Table 2:** Frequency of postoperative opioid use for pain management over time, stratified by treatment arm

Time Period	Frequency, N (%)	Bupivacaine type used in adductor canal block (treatment arm)		
		Liposomal	Conventional	*P*-value
PACU	N	40	40	
	Received opioids	13 (32.5%)	17 (42.5%)	0.49^a^
	Total dose (MME)^b^	8 [7.5–17.5]	7.5 [7.5–17.5]	0.84^c^
POD-0	N	40	39^d^	
	Received opioids	34 (85.0%)	35 (89.7%)	0.74^e^
	Total dose (MME)^b^	26.2 [17.5–49.2]	37.5 [22.5–45.0]	0.016^f^
POD-1	N	37	38	
	Received opioids	30 (81.1%)	35 (92.1%)	0.19^e^
	Total dose (MME)^b^	27.5 [16.9–38.1]	36 [20.0–45.0]	0.016^f^
POD-2	N	36	38	
	Received opioids	24 (66.7%)	34 (89.5%)	0.024^e^
	Total dose (MME)^b^	28.8 [13.1–37.5]	30.0 [20.0–39.8]	0.016^f^

*PACU* post-anesthesia care unit, *POD* post-operative day, *MME* morphine milligram equivalents

^a^Chi square test with continuity correction

^b^Data shown as median [interquartile range]; only those receiving opioids during each specified time period are included

^c^PACU opioid use was compared between study groups by Mann Whitney U test

^d^Pre-discharge opioid data unavailable for *N* = 1

^e^Fisher’s exact test

^f^Repeated measures analysis using a linear mixed effects model after log^10^ transformation of opioid dose data

Table 3 shows that there was no significant difference in pain scores between the two treatment arms, either in the PACU or during POD-0, POD-1 and POD-2. The table also shows that lengths of stay did not differ according to type of local anesthetic used.

**Table 3:** Pain scores over time and postoperative hospitalization metrics, stratified by treatment arm

Variable^a^	Bupivacaine type used in adductor canal block (treatment arm)		
	Liposomal	Conventional	*P*-value
Pain scores over time			
PACU			
N	40	40	
Average pain score	0 [0–3.1]	0 [0–2.0]	0.94^b^
Maximum pain score	0 [0–6.5]	0 [0–4.0]	0.94^b^
POD-0			
N	40	40	
Average pain score	3.2 [2.5–4.4]	3.9 [2.6–4.8]	0.36^b^
Maximum pain score	6 [5–7]	7 [5.1–8]	0.25^b^
POD-1			
N	36	35	
Average pain score	4.5 [3.0–5.7]	5 [3.6–6.0]	0.43^b^
Maximum pain score	6.7 [4–7]	7 [5–7.5]	0.61^b^
POD-2			
N	34	34	
Average pain score	4 [1.9–5.8]	4.2 [3.0–5.6]	0.54^b^
Maximum pain score	4.5 [2–7]	5.5 [4–7]	0.27^b^
Postoperative hospitalization			
Total time in PACU (h)	1.7 ± 0.5	2.0 ± 0.7	0.14^c^
Time, anesthesia start to discharge (h)	25 [7.3–28.9]	23.7 [21.5–27.1]	0.478^b^

*PACU* post-anesthesia care unit, *POD* postoperative day

^a^Data shown as median [interquartile range] or as mean ± standard deviation

^b^Mann Whitney U test

^c^ *t*-test

The supplementary tables provide additional descriptive data. We provide the dose and frequency of non-opioid medications used during the study period as part of multimodal pain management in Table S1. The data indicate that consumption was similar in the two treatment arms. The majority of patients received acetaminophen in both groups at each of the time points evaluated (Table S1). Tables S2, S3 and S4 summarize findings for the per-protocol population, which demonstrated the same overall patterns as those seen in the intention-to-treat population.

## Discussion

Our prospective randomized control trial investigating analgesic differences between LB and CB ACBs for total knee arthroplasty revealed that with LB, opioid use was significantly lower in the days following surgery and that overall total consumption in MMEs was significantly lower from POD0–POD2. There were no differences in pain scores or hospital length of stay. This study has important implications for strategies to improve postoperative outcomes following total knee arthroplasty.

TKA is one of the most common surgeries performed to relieve joint pain in those suffering from knee arthritis [1]. The number of patients requiring total knee arthroplasty has increased markedly over the past two decades [24]. TKA is a procedure associated with moderate-to-severe postoperative pain that affects early ambulation, range of motion, postoperative rehabilitation, patient satisfaction, and patient outcomes [1]. There are reports of worsened post-surgical outcomes when pain is not adequately treated [2, 25]. Identifying optimal strategies to decrease acute pain is crucial for improving post-surgical outcomes in these patients. Furthermore, as with any surgery, reducing postoperative opioid consumption helps to mitigate the risk of opioid misuse [26].

Several prior studies evaluated the effectiveness of perineural LB in improving peripheral nerve block analgesia compared to non-liposomal anesthetics for total knee arthroplasty but the evidence on this topic is not conclusive [17–21]. Yu et al. performed a meta-analysis and found that LB was associated with a reduction in pain scores by 4.22 points 72 h following TKA. However, their assessment was based on a 100-point visual analog scale, inferring that these results were not clinically meaningful. Along similar lines, there were no differences in pain scores at 24 and 48 h, nor were there differences in total opioid consumption and hospital length of stay [19]. On the other hand, Hubler et al., in their prospective randomized trial, found decreased opioid consumption and improved pain scores within the early postoperative period following TKA, but no significant differences in length of hospital stay, patient satisfaction, or adverse postoperative events [21]. However, in a subsequent randomized prospective trial, Hungerford et al. found patients receiving LB for TKA had no improvement in opioid use, length of stay, patient-reported pain, or functional ability compared to those who received a single shot of ropivacaine nerve block [17]. Interestingly, in their prospective trial, Malige et al. observed significantly lower pain scores during the postoperative period but no differences in outpatient opioid consumption [18]. However, it is worth noting that the authors did not specify the overall length of time evaluated during the outpatient period. Furthermore, the authors identified an increased length of stay in the group that did not receive LB, but the length of stay in their study was much longer than that observed in our cohort (36.3 h LB, 49.7 h control group and 23.7–25.8 h in our study). Lastly, Chen et al., in their prospective trial, compared continuous liposomal bupivacaine infusions to liposomal bupivacaine adductor canal blocks. However, their investigation was different from ours since they compared continuous infusions to the single shot liposomal bupivacaine block and a limitation to their study was that 22% of patients in the continuous infusion arm had a catheter-related complication such as early dislodgement and catheter leak. Additionally, their investigation was limited to hospital discharge which was before the 72 h period of liposomal bupivacaine analgesic efficacy which should be evaluated in any study looking at the effectiveness of this medication [27].

While our study was adequately powered to detect a clinically significant difference in pain scores, our data are consistent with those of Hungerford et al. in that we found no significant difference between the treatment arms. We did, however, observe a substantial reduction in opioid utilization during the period evaluated. This objective outcome is particularly important given that the issue of opioid misuse due to opioid overprescribing is a known significant contributor to the opioid epidemic [26]. These same patterns were evident in both the intention-to-treat and per-protocol analyses, suggesting that they are not influenced by any bias introduced by protocol deviations.

Our prospective randomized controlled study design, aimed at minimizing bias, was strengthened by having a single surgeon that performed all operative procedures using the same technique, which helped eliminate procedural performance differences. Conversely, this feature may reduce the generalizability of our results. Additionally, all study staff involved in nerve blocks were board-certified physicians with expertise in block placement, ensuring that any differences between the cohorts were due to the study drug rather than variations in block placement.

Our study focused on the analgesic outcomes associated with liposomal bupivacaine. We acknowledge the limitation that because of this focus, our study did not evaluate other factors related to post-arthroplasty rehabilitation, such as knee range of motion, nor did we explore its influence on postoperative readmission rates. Future studies might investigate the relationship between the enhanced analgesic outcomes and various metrics that serve as indicators for post-arthroplasty rehabilitation progress.

The current cost of a 266 mg vial of LB is $365, significantly higher than the cost of CB which is around $3 [28, 29]. Therefore, the use of LB should be justified by demonstrating its benefits. As noted above, findings have been inconsistent regarding the benefit of LB in improving analgesic outcomes following TKA. As such, various studies have also explored the relationship between the use of LB and overall healthcare costs [30–32]. While these studies have reached different conclusions regarding the analgesic benefits of LB, the cost of LB did not negatively impact the overall hospitalization costs related to surgery. In a large retrospective observational study by Asche et al., TKA procedures that utilized LB were associated with lower total hospitalization costs due to reduced length of stay and increased likelihood of home discharge [30]. However, in a pragmatic randomized clinical trial conducted by Hamilton et al., there were no differences in opioid consumption, functional outcomes, or pain scores within the evaluated 72 h period for patients receiving LB compared to conventional bupivacaine for TKA [31]. Furthermore, there were no significant differences in the total costs of care between the two groups when factoring the cumulative costs of the surgical procedure, associated hospital stay, and administered analgesic medications [31]. These studies suggest that LB does not increase the overall cost of care but its usage should still be based on proven benefits.

## Conclusions

The findings from our own randomized controlled trial provide additional evidence that postoperative blocks using LB in ACBs may not effectively reduce pain after surgery. However, they do support the notion that LB has a positive impact on reducing postoperative opioid consumption, indicating a potential benefit in this aspect of patient care. Future studies are needed to investigate the effectiveness of LB for specific procedural types and to explore potential neuro-anatomical differences that may help explain the inconsistencies in its reported effectiveness.

In conclusion, we found that using LB in an ACB after TKA led to decreased opioid consumption up to 72 h following surgery. These results add to the existing literature regarding the benefits of LB and may inform the current practice of the off-label use of LB after TKA.

### Supplementary Information

**Additional file 1: Supplementary Table 1.** Post-PACU use of non-opioids for pain management, stratified by treatment arm. **Supplementary Table 2.** Frequency of post-operative opioid use for pain management over time; per-protocol analysis. **Supplementary Table 3.** Post-operative opioid dose over time among patients receiving opioids for pain management; per-protocol analysis. **Supplementary Table 4.** Pain scores over time and post-operative hospitalization metrics: per protocol analysis, stratified by treatment arm.

## Data Availability

The datasets used and analyzed during the current study are available from the corresponding author on reasonable request.

## Abbreviations

TKA: Total knee arthroplasty
ACB: Adductor canal nerve blocks
CB: Conventional bupivacaine
LB: Liposomal bupivacaine
MME: Morphine milligram equivalents
POD: Postoperative day
PACU: Post-operative care unit
NRS: Numeric rating scale
