# Supplementary material for: A prospective, randomized trial of liposomal bupivacaine compared to conventional bupivacaine on pain control and postoperative opioid use in adults receiving adductor canal blocks for total knee arthroplasty
Source: Arthroplasty. 2024 Feb 1;6:6. doi: 10.1186/s42836-023-00226-y (PMC10832097; doi:10.1186/s42836-023-00226-y)
Supplement: Supplementary file 1 — Additional file 1: Supplementary Table 1. Post-PACU use of non-opioids for pain management, stratified by treatment arm. Supplementary Table 2. Frequency of post-operative opioid use for pain management over time; per-protocol analysis. Supplementary Table 3. Post-operative opioid dose over time among patients receiving opioids for pain management; per-protocol analysis. Supplementary Table 4. Pain scores over time and post-operative hospitalization metrics: per protocol analysis, stratified by treatment arm. [file 42836_2023_226_MOESM1_ESM.docx]

Supplementary Table 1: Post-PACU use of non-opioids for pain management, stratified by treatment arm.

| Variable | Bupivacaine type used in adductor canal block (treatment arm)^a^ | |
| --- | --- | --- |
|  | Liposomal | Conventional |
| PACU |  |  |
| n | 40 | 40 |
| Received ketoroloac | 5 (12.5) | 10 (25.0) |
| Dose, if received (mg) | 15 (all) | 15 (all) |
| Received PO acetaminophen | 0 (0.0) | 1 (2.5) |
| Dose, if received (mg) | -- | 650 |
| POD-0 |  |  |
| n | 40 | 39 |
| Received ketoroloac | 30 (75.0) | 27 (74.4) |
| Dose, if received (mg) | 44.0 ± 16.7 (15-60) | 44.0 ± 15.5 (15-60) |
| Received PO acetaminophen | 37 (92.5) | 38 (97.4) |
| Dose, if received (mg) | 2480 ± 575 (1950-4000) | 2268 ± 497 (1000-3000) |
| POD-1 |  |  |
| n | 37 | 38 |
| Received ketoroloac | 7 (18.9) | 4 (10.4) |
| Dose, if received (mg) | 16.1 ± 2.8 (15-22.5) | 15.0 (all) |
| n | 37 | 39 |
| Received PO acetaminophen | 36 (97.3) | 38 (97.4) |
| Dose, if received (mg) | 2779 ± 614 (1300-4000) | 2821 ± 646 (650-4000) |
| POD-2 |  |  |
| n | 36 | 38 |
| Received ketoroloac | 0 (0.0) | 0 (0.0) |
| Received PO acetaminophen | 34 (94.4) | 38 (100.0) |
| Dose, if received (mg) | 2798 ± 451 (2000-3900) | 2828 ± 660 (1000-4000) |

Abbreviations: PACU, post-anesthesia care unit; POD, post-operative day; PO, by mouth

^a^ Data shown as n (%) or as mean ± standard deviation (full range)

Supplementary Table 2: Frequency of post-operative opioid use for pain management over time; per-protocol analysis

| Time Period | Frequency, n (%) | Bupivacaine type used in adductor canal block (treatment arm) | |
| --- | --- | --- | --- |
|  |  | Liposomal | Conventional |
| PACU | n | 34 | 32 |
|  | Received opioids | 11 (32.4) | 15 (46.9) |
| POD-0 | n | 34 | 32 |
|  | Received opioids | 28 (84.2) | 28 (87.5) |
| POD-1 | n | 34 | 32 |
|  | Received opioids | 27 (79.4) | 30 (93.8) |
| POD-2 | n | 33 | 32 |
|  | Received opioids | 21 (63.6) | 28 (87.5) |

Abbreviations: PACU, post-anesthesia care unit; POD, post-operative day

Supplementary Table 3: Post-operative opioid dose over time among patients receiving opioids for pain management; per-protocol analysis

| Time period | Opioid consumption^a^ | Bupivacaine type used in adductor canal block (treatment arm) | |
| --- | --- | --- | --- |
|  |  | Liposomal | Conventional |
| PACU | n | 11 | 15 |
|  | Total dose (MME) | 8 [7.5-17.5] | 7.5 [7.5-27.5] |
| POD-0 | n | 28 | 28 |
|  | Total dose (MME) | 26.2 [17.5-44.4] | 40.0 [22.9-57.5] |
| POD-1 | n | 27 | 30 |
|  | Total dose (MME) | 25.0 [15.0-37.5] | 36.8 [19.4-45.0] |
| POD-2 | n | 21 | 28 |
|  | Total dose (MME) | 27.5 [11.2-35.2] | 30.0 [18.1-45.0] |

Abbreviations: PACU, post-anesthesia care unit; MME, morphine milligram equivalents; POD, post-operative day

^a^ Data shown as n (%) or as median [interquartile range]

Supplementary Table 4: Pain scores over time and post-operative hospitalization metrics: per protocol analysis, stratified by treatment arm

| Variable | Bupivacaine type used in adductor canal block (treatment arm)^a^ | |
| --- | --- | --- |
|  | Liposomal | Conventional |
| n | 34 | 32 |
| Pain Scores over time |  |  |
| PACU |  |  |
| n | 34 | 32 |
| Average pain score | 0 [0-3.6] | 0 [0-2.0] |
| Maximum pain score | 0 [0-7] | 0 [0-4.0] |
| POD-0 |  |  |
| n | 34 | 32 |
| Average pain score | 3.3 [2.4-4.6] | 4.0 [2.6-4.8] |
| Maximum pain score | 6 [5.0-7.0] | 7 [5.1-8] |
| POD-1 |  |  |
| n | 31 | 27 |
| Average pain score | 4.5 [2.8-5.2] | 5 [3.4-6.0] |
| Maximum pain score | 6.0 [4.0-7.0] | 6 [5.0-7.0] |
| POD-2 |  |  |
| n | 29 | 28 |
| Average pain score | 3 [1.4-5.4] | 4.1 [3.0-5.8] |
| Maximum pain score | 4 [2-7] | 5 [4-7] |
| Post-operative hospitalization |  |  |
| Total time in PACU (hours) | 1.8 ± 0.6 | 2.1 ± 0.8 |
| Time, anesthesia start to discharge (hours) | 25.1 [7.1-29.1] | 23.3 [20.8-27.0] |

Abbreviations: PACU, post-anesthesia care unit; POD, post-operative day

^a^ Data shown as median [interquartile range] or as mean ± standard deviation
